# Falls in advanced old age: recalled falls and prospective follow-up of over-90-year-olds in the Cambridge City over-75s Cohort study

**DOI:** 10.1186/1471-2318-8-6

**Published:** 2008-03-17

**Authors:** Jane Fleming, Fiona E Matthews, Carol Brayne, the Cambridge City over-75s Cohort (CC75C) study collaboration

**Affiliations:** 1 Department of Public Health and Primary Care, University of Cambridge, Cambridge, UK; 2 Cambridge University Hospitals Addenbrooke's NHS Foundation Trust, Cambridge, UK; 3 Medical Research Council Biostatistics Unit, Cambridge, UK

## Abstract

**Background:**

The "oldest old" are now the fastest growing section of most western populations, yet there are scarcely any data concerning even the common problem of falls amongst the very old. Prospective data collection is encouraged as the most reliable method for researching older people's falls, though in clinical practice guidelines advise taking a history of any recalled falls. This study set out to inform service planning by describing the epidemiology of falls in advanced old age using both retrospectively and prospectively collected falls data.

**Methods:**

***Design***: Re-survey of over-90-year-olds in a longitudinal cohort study – cross-sectional interview and intensive 12-month follow-up.

***Participants and setting***: 90 women and 20 men participating in a population-based cohort (aged 91–105 years, in care-homes and community-dwelling) recruited from representative general practices in Cambridge, UK

***Measurements***: Prospective falls data were collected using fall calendars and telephone follow-up for one year after cross-sectional survey including fall history.

**Results:**

58% were reported to have fallen at least once in the previous year and 60% in the 1-year follow-up. The proportion reported to have fallen more than once was lower using retrospective recall of the past year than prospective reports gathered the following year (34% versus 45%), as were fall rates (1.6 and 2.8 falls/person-year respectively). Repeated falls in the past year were more highly predictive of falls during the following year – IRR 4.7, 95% CI 2.6–8.7 – than just one – IRR 3.6, 95% CI 2.0–6.3, using negative binomial regression. Only 1/5 reportedly did not fall during either the year before or after interview.

**Conclusion:**

Fall rates in this representative sample of over-90-year-olds are even higher than previous reports from octogenarians. Recalled falls last year, particularly repeated falls, strongly predicted falls during follow-up. Similar proportions of people who fell were reported by retrospective and prospective methods covering two consecutive years. Recall methods may underestimate numbers of repeated falls and the extent of recurrent falling. Professionals caring for people of advanced age can easily ask routinely whether someone has fallen at all, or more than once, in the past year to identify those at high risk of subsequent falls.

## Background

The "oldest old" are now the fastest growing section of most western populations. Britain's population aged 85 or older has already almost trebled in three decades (to over 1.1 million people in 2003) and is set to double again within the next quarter-century [1]. With rising numbers of people living to a very old age, information about this growing population is clearly needed but limited in availability.

### Lack of data on falls in older old age

There is a dearth of evidence regarding even the common problem of falls amongst the very old, an information gap that urgently needs filling. Few studies have included more than a small minority of octogenarian participants, even fewer nonagenarians, and falls rates in these age-bands have rarely been presented. Those studies which do report for these age-groups have had very small numbers, for example one reported rate of 152 falls/100-person-years amongst over-90-year-olds [2] had only 2% of their sample at this age on which to base this incidence. The picture of health conditions affecting older old people is different from that for younger old age [3] so simple extrapolation cannot provide adequate estimates on which to plan for future demographic change.

### Methods of measuring falling

How falls data are gathered is important for validity and reliability. There are questions of defining or classifying falls and methodological issues including information sources, ethics and data collection tools. Taking a "fall history" is important in clinical practice and retrospective recall is often used in research data collection to save time and costs. However, the length of recall period used can have considerable effects on the data gathered [4]. Under-reporting of falls is a largely un-quantified problem but has long been an acknowledged limitation with recall methods [5]. A prospective approach has often been regarded as the method of choice in falls research, however fewer than half the studies in a recent systematic review of fall measurement used prospective data collection [6]. The impact on estimates of using more economical methods is largely unknown. This study set out to address these gaps using data from two consecutive years gathered retrospectively and prospectively.

## Methods

### Participants from a population-based cohort

The Cambridge City over-75s Cohort (CC75C) is a longitudinal study of ageing which began in 1985 [7,8]. Participants were initially recruited from socially and geographically representative primary care practices, with a 95% baseline response rate equivalent to 40% of the total over-75-year-old population in Cambridge, UK. The cohort has been followed-up with successive interviews every few years, each approved by Cambridge Research Ethics Committee, with death the principal cause of attrition. As well as the standard CC75C nurse-administered interview, the Year 17 survey added a special focus on falls in advanced old age, including retrospective measures and prospective follow-up (2002–4).

### Measurements

#### Previous falls recalled – prevalence, frequency and time since last fall

At interview respondents were asked "Have you fallen in the last three months?"/"...in the last year?" and as accurate as possible an estimate of the timing of any fall(s) was sought. Information given was coded to provide summary measures of frequency of remembered falls comparable with measurement periods used earlier in the CC75C study and in other studies. Reporting more than one fall in the previous year was classified as repeat falling. Those who reported no falls in the previous year were asked "If you have ever fallen, how long ago was the last time you fell?".

#### Falls during follow-up

Data on falls during the year after interview were collected prospectively for everyone who took part in the latest survey. Follow-up was for twelve months or until death if sooner and involved a combination of methods. Respondents were asked whether they would "be happy to let us know if [they] were unfortunate enough to fall at all any time over the next year". Participants and/or their carers willing and able to provide even minimal information by completing a tear off page and returning it weekly were given fall calendars and pre-paid envelopes. Regular follow-up phone-calls were the alternative method for those participants or proxy informants who were unable or reluctant to complete the calendars, or if no calendar had been received for over a fortnight. If participants were resident in long-term care, they and/or their proxy informants were asked for consent to follow up falls using the institutions' records and care homes were contacted every four weeks. On occasions when repeated attempts to contact by telephone were unsuccessful visits were made in person. Participants were also contacted after reporting a fall, as far as possible in person but also by telephone when this was necessary to avoid delayed contact.

#### Fall definition

The definition of a fall used throughout was that of the Kellogg International Work Group on the Prevention of Falls – "unintentionally coming to the ground or some lower level other than as a consequence of sustaining a violent blow, loss of consciousness, sudden onset of paralysis as in stroke or seizure" [9]. Participants and proxy informants were encouraged to report anything that could be construed as falling, even "nearly" falling, in order to avoid the under-reporting of falls that a respondent might feel "didn't really count as a fall" [10]. All reports were then coded as either a "fall" or a "near fall", according to whether or not they met the definition. It was impossible to achieve blinding in the coding of fall reports but each report underwent two separate reviews for classification as an actual fall or near fall, this repeat process resulting in only one report being re-classified.

### Statistical methods

All analyses were conducted in Stata (Intercooled version 9).

#### "Prevalence" of falling and repeat falling

For both the retrospective and prospective data two sets of cross-tabulations were used to examine as separate outcomes reporting any fall and reporting more than one fall over the year before and after interview respectively – recalled and follow-up "prevalence" of falling and repeat falling. As odds ratios overestimate relative risk for outcomes that are common in the population under study (as falls were in our sample), relative risks were calculated directly where possible. Otherwise – for non-dichotomous variables and for multiple-variable models – relative risks were calculated from logistic regression odds ratios following established methods [11].

#### "Incidence" of falls

Taking account of the data collected on fall frequency – recalled and follow-up falls "incidence" – the number of falls reported in the past year and during the follow-up year were used for two sets of negative binomial regression analyses, the form of Poisson regression recommended for handling the over-dispersion typically found in falls data [12,13]. In all these regressions robust methods were used to calculate conservative confidence intervals.

#### Fall free survival

Kaplan-Meier survival plots of the time from interview until the first fall reported during follow-up and Cox regression hazard ratios were used to describe fall free survival.

#### Multiple-variable analyses

The multi-factorial aetiology of falling in old age is complex and will be further examined with this study sample in a subsequent paper. For this report's focus on descriptive epidemiology analyses of association only examined the potentially confounding effects of socio-demographic co-variates. For each of these, likelihood ratio tests with multi-factorial models for all three regression methods (logistic, negative binomial and Cox regressions) examined the potentially confounding effects of all the other socio-demographic covariates. Adjusted odds ratios from the logistic regressions were converted to relative risks as described above [11].

#### Missing data

Analyses use information from interviews with proxy informants as well as study participants themselves, drawing on whichever source provided more information. Information was available about falls in the year before interview for all but one participant who had recently been moved between residential homes. Another participant remembered falling some time in the last year but could not be more precise, and two further participants were reported to have had no falls in the past year but whether they had fallen before this period was unknown. Thus the denominators shown in the results sections vary slightly between recalled measures. Prospective falls data were collected on all participants: only one respondent's next-of-kin did not want her father or herself bothered with either fall calendars or phone-calls. However, the study was subsequently notified of his death and the falls prompting the hospital admission that preceded it. Years of full-time education and social class data were collected at baseline, the latter missing for n = 4.

## Results

### Characteristics of the sample

Ninety women and 20 men (84% of cohort survivors) were included in the survey: 90% personally took part (n = 99) while proxy informants provided all information for those unable to be interviewed and supplementary information for others (n = 34). Reasons for lack of interview with the participant themselves included failure to trace (n = 3), access denied by a relative or GP (n = 4), illness (n = 4), profound deafness (n = 2) and refusal (19), but proxy informants gave interviews for 11 of these.

Ages ranged from 91 to 105 years old, mean (SD) 94.4 (2.4), with no marked age differences in the sample by gender although the larger numbers of women gave a wider distribution. They were a predominantly frail population – 11% housebound, more than a third severely cognitively impaired, 2/3 with difficulty in at least two basic activities of daily living, 4/5 affected by at least five health conditions, most unable to climb stairs and only a fifth still able to walk around their local neighbourhood. Over half the participants were living in the community, a further sixth lived in sheltered accommodation and a quarter in institutional care, mainly residential homes.

The following findings describe the study's falls measures in terms of how common falling was amongst these over-90-year-olds (the "prevalence" of falling and repeated falling) and how often they fell (incidence of falls) using both retrospective and prospective data plus additionally, from the latter, how long before any fall occurred.

### Falls recalled retrospectively

Well over half the participants (58%) reportedly fell in the year before interview and 59% of them, or 34% of the total, reported falling more than once in the past year. A fifth recalled falling at least three times in the last year, and three people a dozen or more times.

**Figure 1:**
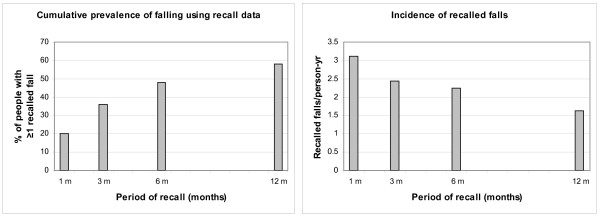
**Retrospective prevalence and incidence of falling – effect of recall period length**. The longer the length of time about which respondents are questioned regarding their fall history the more prevalent falling appears. By contrast, calculating falls incidence using recalled falls gives higher incidence rates with shorter recall periods.

Even more of the study participants were reported to have fallen at least once in the past five years than in the past year (88% vs 58%). Figures 1a and 1b illustrate how the prevalence of falling, based on reports of remembered falls, increases with the length of time about which respondents are questioned regarding their fall history. However, by contrast, incidence rates based on of the number of falls reported as having happened within different time periods before interview increase with shorter recall periods from 1.6 falls/person-year taking the full previous year to 2.2, 2.4 and 3.1/person-year in the past 6, 3 and 1 months respectively.

Comparison of the time since the participants were said to have last fallen and the time since they said they last hurt themselves falling suggest that falls resulting in injury appear to be better recalled, with a longer median time to the last remembered injurious fall (9 months, IQR 2–30 months) than to any last fall (5.5 months, IQR 1–23 months).

In univariate analyses of association with socio-demographic factors (see Table 1) increasing age, higher educational level and living in any sheltered scheme or care home were all associated with having fallen in the past year, as recalled by participants and proxy informants. Only age was associated with recalled repeated falls – two or more falls in the year before interview. Regressions using the number of falls recalled as having occurred in the previous year also showed older age, even across such a limited age range, and living in any supported setting increased falls risk ratios. Almost identical proportions of both men and women remembered having fallen in the past year (55% and 58% respectively); differences widened in repeated falling measures with falls rates 50% higher in women, but these differences did not reach significance. Multiple variable regressions in these retrospective data modelled the effects on each socio-demographic variable of the others. Age was the only factor that remained significantly associated with increased risk of falls (Each year older than 91: IRR 1.1, 95% C.I. 1.0–1.3; Ageband ≥ 95 vs ≤ 94: IRR 1.8, 95% C.I. 1.0–2.9) and with repeated falling (Each year older than 91: RR 1.3, 95% C.I. 1.1–1.6; Ageband ≥ 95 vs ≤ 94: RR 2.8, 95% C.I. 1.7–3.6) when adjusted for other demographic covariates. However, place of residence (RR 1.4, 95% C.I. 1.0–1.8) and educational level (RR 1.5, 95% C.I. 1.0–1.7) as well as age (Each year older than 91: RR 1.1, 95% C.I. 1.0–1.2; Ageband ≥ 95 vs ≤ 94: RR 1.5, 95% C.I. 1.1–1.8) continued to be associated with having suffered any fall in the past year (adjusted risk estimates not tabulated).

**Table 1:** Prevalence, incidence and risk estimates of remembered falls in the previous year

**Study participant characteristics**		**Prevalence: ** **How common is falling or repeated falling** ** >90 years?**				**Incidence: ** **How frequently do** ** >90-year-olds fall?**			
	n	Recalled ≥ 1 fall in past year n (%)	Relative Risk – unadjusted RR (95% C.I.)	Recalled ≥ 2 falls in past year n (%)	Relative Risk – unadjusted RR (95% C.I.)	Number of recalled falls in past year	Person- yrs of recalled falls data	Incidence of recalled falls/person-yr	Incidence Rate Ratio – unadjusted IRR (95% C.I.)

**AGE**									
Each additional year > 91 years			**1.1 (1.0–1.2)**		**1.3 (1.1–1.5)**	-	-	-	**1.1 (1.0–1.3)**
**AGE-BAND**									
91–94 years old	74	36 (48)	1.0	16 (22)	1.0	94	74	1.3	1.0
≥ 95 years old	36	27 (77)	**1.6 (1.2–1.9)**	21 (60)	**2.7 (1.8–3.6)**	83	35	2.4	**1.9 (1.1–3.3)**
**GENDER**									
Men	20	11 (55)	1.0	5 (25)	1.0	23	20	1.2	1.0
Women	90	52 (58)	1.1 (0.6–1.4)	32 (36)	1.4 (0.7–2.5)	154	89	1.7	1.5 (0.8–3.0)
**PLACE OF RESIDENCE**									
Living in the community (house, flat or granny flat)	62	30 (48)	1.0	18 (29)	1.0	74	62	1.2	1.0
Living in any supported setting (Sheltered housing or institution)	48	33 (70)	**1.5 (1.1–1.8)**	19 (40)	1.4 (0.9–2.1)	103	47	2.2	**1.8 (1.1–3.2)**
**EDUCATION**									
Left school aged 14 yrs or less	67	33 (50)	1.0	20 (30)	1.0	91	66	1.4	1.0
Full-time education aged 15+ yrs	43	30 (70)	**1.4 (1.0–1.7)**	17 (40)	1.3 (0.8–2.0)	86	43	2.0	1.5 (0.8–2.6)
**SOCIAL CLASS**									
Manual	55	30 (55)	1.0	20 (36)	1.0	72	55	1.3	1.0
Non-manual	51	30 (60)	1.1 (0.8–1.4)	15 (30)	0.9 (0.4–1.4)	96	50	1.9	1.5 (0.8–2.6)

TOTAL SAMPLE	110	63 (58)		37 (34)		177	109	1.6	

### Falls reported during prospective follow-up

#### Length of follow-up

Twelve months' follow-up was completed for 75% of participants (n = 82) but 6 men (30%) and 22 women (24%) died within a year of being interviewed. The total follow-up period was 95.7 person-years, range 2–52 weeks, mean (SD) 45 (14) weeks.

#### Falls, "near falls" and "non follow-up falls"

In all 290 reports were made of incidents that might be counted as falls during follow-up. Three had to be discounted, two because they occurred outside the follow-up period and a third because a neighbour's report of participant's broken leg turned out to be due to a pathological fracture without any fall. Of the remainder, only 22 were classified as "near falls" for failure to meet the Kellogg definition [9], leaving 265 valid fall reports within the follow-up.

#### Prevalence, incidence and fall-free survival

**Figure 2:**
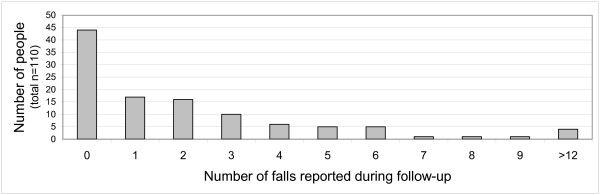
**Reported numbers of falls during follow-up**. The distribution of falls reported during follow-up was wide-ranging: only a quarter of those who reported falls had only one.

These reports of falls during follow-up affected 66 individuals (10 men and 56 women), a prevalence rate of 60%. Falling more than once was also very common: 7 men and 42 women fell at least twice. Thus three-quarters of the "fallers", or 45% of the full sample, were "repeat fallers". Half of those who fell reported three or more falls: Figure 2 illustrates the wide distribution. These 265 falls occurring over 95.7 person-years of follow-up give an incidence rate of 2.8 falls per person-year, median (IQR) 2.5 (1 – 5). Mean time to first fall was 111 days; a quarter had suffered at least one fall by 62 days from interview and half within 181 days.

Table 2 tabulates the prevalence and incidence of these prospectively reported falls by demographic characteristics alongside the associated risk estimates, including hazard ratios from fall-free survival analysis. The effects of increasing age on falling was less than in the retrospective data. Proportionately more women than men fell during follow-up: a quarter more fell at least once and a third more women than men fell repeatedly but, conversely, the prospectively calculated falls rate was slightly lower amongst women than men. Proportions who fell and numbers of falls were both higher amongst people living in supported settings, in fact slightly higher in sheltered accommodation than in care homes (3.7 and 2.8 falls/person-year respectively – not tabulated). Non-manual social class was associated with higher fall rates, the only factor besides age for which any risk estimates reached significance. Each year older remained a just significant predictor of slightly rising fall rates when adjusted for other socio-demographic factors (IRR 1.06, 95% C.I. 1.0–1.2), but adjusting for these co-variates removed the effect of social class, and did not reveal any significant effect of either gender or residential setting on fall rates. Education beyond the minimum school leaving age appeared to predict falls when adjusted for age, sex and place of residence (IRR 1.9, 95% C.I. 1.1–3.4).

**Table 2:** Prevalence, incidence, fall free survival and risk estimates of prospectively reported falls during follow-up year

**Study participant characteristics**		**Prevalence:** **How common is falling or repeated falling ** **>90 years?**				**Incidence:** **How frequently do ** **>90-year-olds fall?**				**Fall free survival:** **How long till a fall?**	
	n	Reported ≥ 1 fall in follow/up n (%)	Relative Risk – unadjusted RR (95% C.I.)	Reported ≥ 2 falls in follow/up n (%)	Relative Risk – unadjusted RR (95% C.I.)	N° of f/up falls	P-yrs of f/up	F/up falls /p-yr	Incidence Rate Ratio – unadjusted IRR (95% C.I.)	Time to 1^st ^fall Mean (SD)	Hazard Ratio -unadjusted HR (95% C.I.)

**AGE**											
Each additional year > 91 years		-	**1.0 (1.0–1.1)**	-	**1.1 (1.0–1.2)**	-	-	-	**1.04 (1.0–1.2)**		-
**AGE-BAND**											
91–94 years old	74	43 (58)	1.0	31 (42)	1.0	174	68.2	2.6	1.0	130(89)	1.0
≥ 95 years old	36	23 (64)	1.1 (0.8–1.4)	18 (50)	1.2 (0.7–1.7)	91	27.5	3.3	1.5 (0.7–3.1)	75(66)	1.6 (0.9–2.6)
**GENDER**											
Men	20	10 (50)	1.0	7 (35)	1.0	54	17.2	3.1	1.0	108(92)	1.0
Women	90	56 (62)	1.3 (0.8–1.6)	42 (47)	1.3 (0.7–2.0)	211	78.5	2.7	0.8 (0.3–2.3)	111(85)	1.3 (0.7–2.6)
**PLACE OF RESIDENCE**											
Living in the community (house, flat or granny flat)	62	35 (57)	1.0	25 (40)	1.0	138	56.1	2.5	1.0	116(96)	1.0
Living in any supported setting (Sheltered housing or institution)	48	31 (65)	1.1 (0.8–1.4)	24 (50)	1.3 (0.8–1.7)	127	39.7	3.2	1.4 (0.8–2.4)	104(73)	1.3 (0.8–2.1)
**EDUCATION**											
Left school aged 14 yrs or less	67	38 (57)	1.0	26 (39)	1.0	118	57.1	2.1	1.0	126(95)	1.0
Full-time education aged 15+ yrs	43	28 (65)	1.1 (0.8–1.4)	23 (54)	1.4 (0.9–1.8)	147	38.6	3.8	1.8 (0.9–3.7)	89(66)	1.4 (0.8–2.2)
**SOCIAL CLASS**											
Manual	55	34 (62)	1.0	21 (38)	1.0	95	46.5	2.0	1.0	120(86)	1.0
Non-manual	51	30 (59)	1.0 (0.6–1.2)	27 (53)	1.4 (0.9–1.9)	163	46.2	3.5	**1.9 (1.0–3.6)**	103(87)	0.9 (0.5–1.5)
**FALL HISTORY**											
Not fallen in year before interview	46	23 (50)	1.0	14 (30)	1.0	47	40.8	1.2	1.0	132(92)	1.0
Fallen at least once in past year	63	42 (67)	1.3 (0.9–1.6)	34 (54)	**1.8 (1.1–2.4)**	216	54.6	4.0	**3.6 (2.0–6.3)**	101(81)	**1.7 (1.0–2.8)**
Fallen only once or not past year	72	32 (44)	1.0	21 (29)	1.0	80	62.4	1.3	1.0	137(89)	1.0
Fallen > once in past year	37	33 (89)	**2.0 (1.6–2.2)**	27 (73)	**2.5 (1.8–3.0)**	183	33.0	5.6	**4.7 (2.6–8.7)**	88(75)	**3.5 (2.1–5.7)**

TOTAL SAMPLE	110	66 (60)		49 (45)		265	95.7	2.8		111(85)	

#### Repeated falling

**Figure 3:**
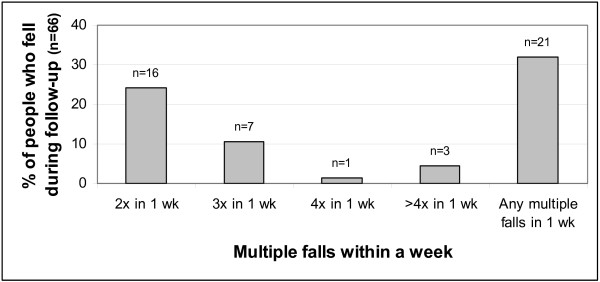
**Episodes of multiple falls reported during follow-up: proportions of people that fell who reported >1 fall within a week**. Almost 1 in 3 of those who fell during follow-up fell more than once within a week, this total lower than the sum of each category shown because some people suffered such multiple fall episodes more than once.

Many of the repeated falls occurred within a short time frame of each other. Figure 3 shows the proportions of participants who fell that reported falling 2, 3, 4 or more times in any week during follow-up: 21 people (almost a third of those who fell at all, almost a fifth of the full sample) suffered such episodes of multiple falls within a week, some of them in more than one week. Half those who fell twice within a week had falls on two consecutive days, and five people fell twice on the same day on at least one occasion, in all 10 people who fell more than once in two days.

#### Recalled falls as predictors of subsequent falls

Only 23/110 (21%) neither recalled any falls in the previous year nor fell during follow-up. Analyses explored separately the effects of recalling any fall and repeated falls last year, given that the latter identifies a group potentially at higher risk than the former broader category.

**Figure 4:**
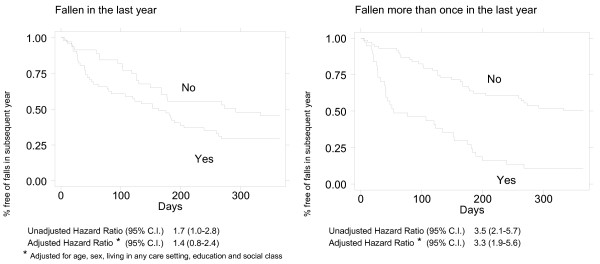
**Time to first fall during follow-up by remembered previous fall history**. There was widening in divergence fall-free survival time between people with any recalled falls in the past year and those with none (Figure 4a) and even more divergence between those with or without repeated recalled falls (Figure 4b).

Figure 4's Kaplan-Meier plots of how falls in the previous year relate to falling during follow-up illustrate the widening disparity in time to first fall between people with any recalled falls and those with none (Figure 4a) and, even more so, between those with or without repeated falls (Figure 4b) as recalled in the past year. These and all Table 2's risk estimates reflect the greater effect of repeated falls. Risk ratios for suffering any fall or more than one fall during follow-up were barely affected by adjusting for socio-demographic covariates. Adjustment for these confounders slightly reduced the strength of both the relative risk and hazard ratio associated with having suffered any fall in the past year (IRR 2.9, 95% C.I. 1.7–5.0; HR 1.4, 95% C.I. 0.8–2.4), but the incidence rate ratios associated with repeated recalled falls remained similar or stronger (IRR 5.0, 95% C.I. 2.8–8.9; HR 3.3, 95% C.I. 1.9–5.6).

## Discussion

Intensive prospective data collection following-up all participants in the 2002–3 CC75C survey found an incidence of 2.8 falls/person-year. Of these over-90-year-old men and women 60% fell at least once in the year after interview, closely matching the proportion who remembered falling in the year before interview (58%). Three-quarters of the study participants who fell, or 45% of the full sample, had more than one fall during follow-up, but only a third reported having fallen more than once in the previous year.

These results are from reports by retrospective and prospective methods covering two consecutive years, thus direct comparison is not intended. Some variation between years would be expected: not only might individual health histories have changed or high risk individuals may have been identified for a preventive intervention, but the sample overall was older during the year after interview than the preceding year, with mortality also potentially affecting findings. However, the proportion of people who fell according to remembered fall history is strikingly similar to that observed during intensive follow-up. By contrast, the recalled falls may under-estimate the extent of repeat falling.

### Strengths and limitations of the study

This unique cohort remains a highly representative population-based sample, using systematic tracing and careful re-recruitment to ensure a low drop-out rate: for only 16% of living survivors was it not possible to obtain even proxy informant data. No other study to date has gathered prospective falls data specifically from older people of such advanced age, representative of their population base. Intensive 12 months' follow-up covered the full sample, with methods that proved to be feasible and well accepted. Indeed there was great willingness from participants, as well as both formal and informal carers, to help with fall reporting – an indication of the relevance and high importance attached to the problem of falling amongst older people. Building on a long-standing study with the methodology already in place clearly imposes some limitations: the sample size in this survey was pre-determined by the survival of the cohort, though participation still exceeded numbers in the "90 plus" age range included in many larger population studies. Interpretation of the significance or non-significance of the relationships examined between group characteristics and measures of falling requires caution given the small sample size.

### Potential under-reporting of falls

Falls follow-up methods were intensive with phone-calls to cover those who missed returning one of the weekly calendar pages or who preferred telephone reports. Nevertheless, the possibility that there was under-reporting of some falls cannot be ruled out. Over-reporting is far less likely as details recorded ensured that it was soon detected if the same fall was mentioned twice. If there was marked under-reporting, the prevalence of falling could be even more widespread than found and falls incidence rates even higher.

Recall bias can affect any study; it is an anticipated problem in falls studies[5]. Moreover, in this study's age-group cognitive impairment is a likely reason for forgetting falls [4]. The most cognitively impaired in this study all had proxy informants reporting on their behalf, but these proxies may not always have been aware of every fall. There is more scope for under-reporting from those with milder impairment, not all of whom had a proxy source.

### Period of reporting effects on fall estimates

It has long been known that the proportion of people who report past falls varies with the length of recall period questioned, with shorter intervals not necessarily providing the most accurate recall [5]. Our survey gathered data in a format that allowed measurement of different recall time periods and also recorded time lapsed since the last recalled fall. This longer time frame revealed that a fall within the previous five years was remembered for 87% of the participants. As expected, the percentage remembering falls rose with longer time intervals but, as found in the EVOS study [14,15], prevalence does not increase in proportion to length of recall period. This can be interpreted in different ways. It is plausible that falls which happened longer ago are less likely to be remembered, particularly non-injurious falls. Certainly in the current study the time lapsed since a "near fall" was generally short, suggesting these were often dismissed as unimportant and soon forgotten. Moreover falls that had resulted in injury were remembered as having happened longer ago than falls in general. On the other hand, it could be that people remember falls as having happened more recently than they actually did. The rising prevalence of falling and decreasing incidence of falls over longer recall periods shown in Figure 1 may help explain some of the differences found for some factors between the relative risks for falling or repeated falling and the incidence rate ratios for falls (see Tables 1 and 2).

Recall interval is also relevant when comparing falls reported from previous prospective studies that used different systems to record falls. A Japanese study illustrated how differential follow-up methods affected reporting: the prevalence of falling during a year in three comparable groups of men asked about their falls every month, every three months and just once at year end was 21%, 16% and 6% respectively, though no such pattern was found amongst women (26%, 18% and 21% respectively) [16]. However, this gender distinction was not found in CC75C.

### Consistency of reporting falling

A recent systematic review of falls monitoring [4] took prospective data collection using on-going weekly or monthly calendars as their recommended criterion standard, though the authors found insufficient evidence to advise what time interval was optimal. This review concluded that recall methods could be highly specific (91–95%) but less sensitive (80–89%) than prospective. The broad concordance between retrospective and prospective findings shown in the few studies mentioned below, and close agreement found in the current CC75C survey, would fit these conclusions. However, even where similar proportions of people falling are reported for pre- and post-interview periods, these will not necessarily represent the same individuals. The Gloucestershire Longitudinal Study of Disability [17] reported that, across three years of general practitioner checks on over-75-year-olds' health and disability, falling was the most inconsistent measure annually: about 70% of people who had reported a fall in the previous three months no longer reported recent falls at the following year's interview, while 11% became new reporters of falling. Such discrepancies are particularly large for shorter recall times but the same point also applies to longer periods.

### Effects of multiple falls on fall estimates

All risk ratios presented for falls adjust for clustering of some falls, based on the assumption that each participant's falls are not necessarily independent of each other. Although it could be argued that episodes involving a series of falls (perhaps attributable to a common factor) might unduly affect interpretation of the data, 32% of those who fell reported at least two falls in close proximity. Thus such reports cannot be discounted without grossly underestimating both prevalence and incidence rates. However, it is also possible that outlying fall frequency counts may inflate findings, therefore sensitivity analyses examined the effects of excluding multiple falls by different cut-points illustrated in Figure 3. For example three people fell more than four times within a week, each imminently preceding either death or hospital admission. Excluding either all three or just the multiple falls preceding death slightly reduced incidence rates (2.3 and 2.6 falls/person-year respectively). However, it is also arguable that such acute illness or end-of-life falls are not untypical in this advanced age-group and so should be included.

### Effects of mortality on fall estimates

Analyses included four people who died within 8 weeks of interview, all with no reported falls, contributing a total of only 0.3 person-years of prospective follow-up. Although incidence rates are clearly unaffected, this approach may under-estimate prevalence because the true denominator was lower for most of the follow-up.

### Comparison with previous reports

Few other studies have reported fall prevalence specific to the oldest old. The two "old old" studies that have reported falls, with recalled data only, each found very similar proportions had fallen at least once in the year before interview: 45% of the men and women in the Umeå 85+ study[18], and 44–49% of women in two Leiden 85-plus Study interviews [19]. Thus the CC75C findings are between a fifth and a third higher than recorded in these slightly younger cohorts. Cross-sectional and prospective studies of broader age-ranges of older people that have presented age-specific results report the proportion who fall each year as between 35% and 51% of people aged 85 or more [19-22], and between 29% and 41% of people aged 80 or older [21,23-29] except in one small volunteer study that reported annual fall prevalence as 58% based on only 12 individuals aged over 80[30,31].

Estimates of repeated falling – more than one fall in a year – range from 14% to 29% in the two studies that have reported these proportions for age-bands over 80-years-old [24,25], even the higher figure only two-thirds of the proportion of recurrent fallers in CC75C.

Few studies have reported both retrospective and prospective falls data from the same sample, but one study in a younger old sample that also report recalled and followed-up falls from two consecutive years showed a similar trend to ours in reporting more repeated falls in follow-up, but also more single falls. This was the Australian Randwick Falls and Fractures study which found higher fall prevalence amongst women aged ≥ 65 years in follow-up than recall: 38% vs 20% for any falls, 21% vs. 14% for repeated falls [32,33]. A Dutch study of over-70-year-old general practice patients reported falls from different length periods before and after interview: 33% fell at least once during just 36-week's follow-up, a marginally higher proportion than could be expected from the 41% with any fall history in the year before interview, but the repeated falls were in similar proportions (16% in follow-up vs 26% recalled) [34].

Annual fall incidence rates have also rarely been reported for very old people. The Montreal study reported identical rates for men and women aged 80 or older (65.9 falls/100 person-years) [24], notably lower than in New Zealand's Dunedin study[2,35] which did not report sex-specific rates but broke down their over-80-year-olds into three age-bands: incidence rose from 94 to 152 falls/100 person-years between the 80–84 years and over-90s age-bands. Although methodological differences may mean studies are not directly comparable, this puts the CC75C incidence at over 80% higher than the previously reported rate for nonagenarians.

### Previous falls: "one-off" falls and recurrent falling

Having fallen before has been identified repeatedly as a risk factor for falling again, both in major community-based studies [31,34-40] and in institutional settings [41-43]. It has also been suggested that characteristics and risk profiles of people who fall repeatedly are different from those who report "just a one-off" fall [44,45] and diverse studies have presented their findings in terms of which factors identify this higher risk group [23,28,29,33,46,47]. Although not all studies confirm this supposition [31,48], it has been suggested that "once only fallers" have more in common with "non-fallers" than "twice or more fallers" [49]. Amongst the over-90-year-olds in the CC75C study, retrospectively gathered fall reports appeared highly predictive of falls in prospective follow-up. Moreover, in this study sample, recalling more that one fall in the past year strengthened the risk estimates for subsequent falling and recurrent falling associated with any recalled fall in the past year. Adjustment for the potentially confounding effects of socio-demographic variables had minimal effects. Fuller examination of other potential risk factors with this study data [50] is beyond the scope of this report's focus on basic demographic descriptors of falls epidemiology in advanced old age. Falling is a multi-factorial problem and the analyses presented here have not attempted to further un-ravel factors that predict and perhaps pre-date any recalled falls. When asking about previous falls, it is important to also consider factors that contribute to this history of falling and thus also to future risk.

## Conclusion

The CC75C study's findings add important new epidemiological data on the "oldest old" to what is already known about falling in old age. The research reveals both prevalence and incidence amongst over-90-year-olds are exceedingly high, whether recorded retrospectively or prospectively, and recurrent falling was far more common than previous reports suggest. These findings reinforce the important need to identify falling with a view to prevention of further falls.

## Competing interests

The author(s) declare that they have no competing interests.

## Authors' contributions

JF contributed to the study design for the current phase, carried out the fieldwork and analyses reported in this paper, drafted the original paper and approved the final manuscript. CB was one of those responsible for the study's conception and design, had over-all supervisory responsibility for the study, contributed to revisions of the drafted paper and has approved the final manuscript. Current members of the CC75C management committee have also been involved in the study's conception and design (FH, GP), given statistical advise (FM), commented on earlier drafts (MF, CB) and all authors have read and approved the final version of the manuscript.

## Pre-publication history

The pre-publication history for this paper can be accessed here:


